# Vitamin C enhances corneal fungal infection treatment in mice via chemotaxis and anti-inflammation

**DOI:** 10.1128/aac.01165-25

**Published:** 2025-11-28

**Authors:** Yanting Xie, Shoujun Jian, Juan Yue, Chunmei Wang, Junlu Dong, Siyu He, Susu liu, Liya Wang, Hongmin Zhang

**Affiliations:** 1 Department of Ophthalmology, Henan Key Laboratory of Ophthalmology and Visual Science, Henan Provincial People’s Hospital, Henan Eye Hospital, People’s Hospital of Zhengzhou University, Zhengzhou, Henan, China; University Children's Hospital Münster, Münster, Germany

**Keywords:** vitamin C, fungal keratitis, neutrophil infiltration, macrophage, mast cells

## Abstract

Fungal keratitis (FK) is a persistent and vision-threatening disease. The potential of vitamin C (VC) in tissue protection is well-recognized; however, its specific role in FK and its interaction with antifungal drugs are not well defined. In this study, we investigated the role of VC in FK *in vitro* and *in vivo*. It revealed that VC substantially decreases clinical scores and corneal perforation rates in a murine model of FK. VC promoted a concentration-dependent increase in neutrophil infiltration via mast cells *in vitro* and confirmed this effect in *in vivo* experiments. Moreover, VC enhanced fungicidal activity by boosting infiltrated neutrophils, without influencing reactive oxygen species (ROS) production or neutrophil extracellular traps (NETs) formation. VC also mitigated the cytokine storm within bone marrow-derived macrophages and increased neutrophil apoptosis, thereby facilitating the efficient clearance of senescent neutrophils. The combination of VC with amphotericin B (AmB) demonstrated additive antifungal effects, reducing fungal load and corneal perforation. These results indicate that VC is pivotal in defending against fungal corneal infections by promoting neutrophil chemotaxis through mast cells (MCs) and modulating the inflammatory response, without suppressing neutrophils’ antifungal capability. The combination of VC with AmB may present a new therapeutic avenue for FK.

## INTRODUCTION

Fungal keratitis (FK) is a serious ocular condition, characterized by high corneal blindness resulting from fungal infections, presenting a significant challenge to ophthalmologists (1–4). This condition frequently arises following corneal trauma, particularly in agricultural environments, and is predominantly caused by filamentous fungi, such as *Fusarium* and *Aspergillus* species, as well as yeast-like *Candida* (3). Current therapeutic approaches, which include topical and systemic antifungal agents, have achieved limited success, as evidenced by low cure rates and severe complications, such as corneal perforation and enucleation (1, 3, 5, 6). Consequently, there is an urgent need to investigate novel therapeutic strategies to improve clinical outcomes and reduce the incidence of vision loss (7).

Vitamin C (VC) is a well-known antioxidant with a wide range of biological functions, notably in modulating immune responses and enhancing the activity of neutrophils, which are key components of the innate immune system (8–10). Neutrophil chemotaxis is closely linked to mast cells (MCs) (11). The recruited neutrophils are pivotal in combating fungal infections through mechanisms such as the production of reactive oxygen species (ROS) and neutrophil extracellular traps (NETs) (10, 12). Recent studies have intriguingly highlighted the potential antimicrobial properties of VC, demonstrating its efficacy against bacterial pathogens, such as *Escherichia coli* (13) and *Shigella* (2). Nonetheless, the role of VC in FK and its potential to modulate neutrophil function in this context remains largely unexplored. Macrophages are also essential components of the immune response to FK (14). They detect fungal pathogens via pattern recognition receptors, induce pro-inflammatory responses, and are pivotal in resolving inflammation and clearing neutrophils during the later stages of FK (15, 16). In the context of antibacterial infections, VC has been shown to attenuate macrophage activation and reduce the production of inflammatory cytokines, thereby mitigating sepsis (17, 18). However, the influence of VC on macrophage function during fungal infections remains inadequately understood.

In this study, we sought to examine the protective effects of VC in a murine model of FK and to elucidate its mechanisms of action. We specifically hypothesized that VC could enhance neutrophil recruitment and fungicidal activity, thereby mitigating the severity of FK. VC may attenuate inflammatory responses by modulating macrophage inflammatory reactions and promoting neutrophil apoptosis. We also investigated the potential synergistic effects of VC when used in conjunction with conventional antifungal agents, such as amphotericin B, to assess whether this combination could provide additional therapeutic benefits. Our research aims to provide novel insights into the therapeutic potential of VC in the treatment of FK and its capacity to augment existing antifungal therapies.

## MATERIALS AND METHODS

### Fungus strain, growth conditions, and spore extraction

#### Strains

*Fusarium solani* (*F. solani, F.3.5840,* and *F.3.1791*) were obtained from the China General Microbiological Culture Collection Center. *Candida albicans* and *Candida parapsilosis* were isolated from patients with FK at the Henan Eye Institute outpatient department and stored in the ophthalmic microbiology laboratory.

#### Culture

The fungi were cultivated on potato dextrose agar (PDA, #HB0233-12, HoPe Bio-Technology) plates at 28°C (*F. solani*) or 35°C (*Candida*).

#### Spore extraction

During the spore extraction process, the surface of fungi, cultured for a period of 3 to 7 days for *F. solani* strains and 24 hours for *Candida* strains on PDA, was rinsed and scratched with saline solution using a sterile disposable pipette. The resulting suspension was filtered through four layers of sterile gauze to obtain the spore suspension. This suspension was subsequently centrifuged at 300 g at room temperature for 5 minutes and then resuspended in 1 mL of medium. The spore density was quantified using a hemocytometer, and the suspension was prepared for further use. If inactivation was necessary, the spore suspension was subjected to heating in a water bath at 95°C for 15 minutes, and then stored at room temperature for subsequent application.

#### Strain utilization

The *F. solani* strain *F.3.5840* was employed in all *in vivo* and *in vitro* experiments, with the exception of the minimum inhibitory concentration (MIC) and fractional inhibitory concentration (FIC) assays, which incorporated all four strains.

### Animals, experimental design, and methods

#### Animals

Experiments were conducted on male C57BL/6J mice (6–12 weeks old) from SKBEX biology (China). The mice were housed in a temperature-controlled room at 25°C on a 12-hour light/dark cycle with free access to food and water, unless specified otherwise.

#### Vitamin C administration

Based on daily VC needs (0.33 g/L) (19), mice of the VC group were administered orally at 3.0 g/L (equivalent to 0.5 g/kg/day) (20) and adjusted to pH 7.0–7.4 with 1 M NaOH for stability with fresh water provided daily. Other groups without VC received untreated fresh water.

#### Fungal keratitis murine model establishment

Mice were anesthetized with 1% (m/vol) pentobarbital sodium. The right eyes of the mice were used for the FK model following previously described methods (21). A sterile knife made a cross scratch on the central cornea with a depth to reach the stroma. *F. solani* hyphae (strain 3.5840, cultured on PDA PDA for 3–7 days at 28°C) on bamboo sticks were applied to the scratch to simulate natural fungal infection.

#### Experimental grouping

VC effect on FK: mice were randomly divided into wild-type (WT) and VC groups. The WT group received a standard diet and water; the VC group received 3 g/L of VC in water for 7 days before and 72 hours after the FK model (daily replenishment).

VC and mast cell membrane stabilizer (MC-Block) interaction: mice were divided into four groups (n ≧ 6 per group): WT, VC, mast cell membrane stabilized (MC-Block), and mast cell membrane stabilized + VC (MC-Block + VC) group. The WT group and the VC group were described as above. The MC-Block group received an intraperitoneal injection of sodium cromolyn (100 mg/kg; #MB1067, Meilunbio) as described (21). The MC-Block + VC group was given water with pH-adjusted 3 g/L of VC and an intraperitoneal injection of sodium cromolyn as above.

Synergy of VC with amphotericin B (AmB): mice were divided into four groups (n ≧ 6 per group): WT, VC, AmB, and VC + AmB. The WT group had a standard diet, clean water, and received saline eye drops hourly for 12 times a day after fungal exposure for 24 hours. The VC group was given water with 3 g/L VC and saline eye drops. The VC + AmB group received 3 g/L VC water and 0.5% AmB eye drops hourly for 12 times a day. All four groups of mice were treated as the context above for the FK model established.

All experiments were conducted at least three times.

### Clinical measurements of FK and detection of corneal fungal load

#### Clinical scoring

Corneas were continuously observed and photographed at the corresponding time points (0, 12, 24, 48, or 72 hours) under a slit-lamp microscope (SLM-3ER, Kanghuaruiming, China). Clinical scores of the cornea were assessed as described (22) in a single-blinded manner to reduce subjective bias. Perforated eyes or not were also recorded under the slit-lamp microscope, and the depth of hypopyon was measured by the EyeStudio software (V1.0.1).

#### Corneal fungal load

Mice were euthanized at 72 hours after FK introduction, and their corneas were excised, trimmed, and placed in 200 µL of 0.01 M PBS. After mincing with microscissors, the tissues were homogenized at 60 Hz for 60 seconds. The homogenate was fourfold diluted in sterile 0.01 M PBS, and 100 µL was spread onto a PDA plate. The plates were incubated at 28°C in a fungal incubator for 36 to 48 hours. Following incubation at 28°C for 36–48 hours, fungal colonies were photographed and quantified using ImageJ (v1.54f; NIH).

### Isolation and culture of bone marrow-derived cells

#### Bone marrow harvest

Bone marrow cells for our *in vitro* studies were obtained from untreated wild-type male mice with free access to water. The mice were euthanized with 1% sodium pentobarbital, and femurs and tibias were isolated after sterilizing the skin with 75% alcohol. Bone marrow was flushed with saline, filtered through a 100 µm strainer, and centrifuged at 300 × *g* for 5 minutes to collect the cells.

#### Neutrophils

Bone marrow cells collected from 8 to 12-week-old mice were resuspended in 1 mL of 0.01 M PBS. Erythrocytes were lysed through the hypotonic method, and the white blood cells were collected by centrifugation (300 × *g* for 5 minutes). Neutrophils were purified using a discontinuous Percoll gradient (#17089109, Cytiva) and resuspended in RPMI1640 at room temperature used within 2 hours. Neutrophil purity (≥97%) was confirmed using a BD FACSCanto Plus flow cytometer using CD11b (clone M1/70; #45-0112-82, Invitrogen) and Ly6G/6C (clone RB6-8C5; #11-5931-86, Invitrogen) antibodies.

#### Mast cells

As previously described (23), the bone marrow-derived cells were cultured from 6 to 8-week-old mice in 40 mL of mast cell medium (RPMI 1640 medium supplemented with 10% fetal bovine serum, 100 U/mL penicillin/streptomycin, 4 mM L-glutamine, 1 mM sodium pyruvate, 1% MEM nonessential amino acids, 50 µM 2-mercaptoethanol, and 10 µg/L IL-3 [#213-13, PeproTech]) at 37°C with 5% CO_2_ for 4–6 weeks. Medium (40 mL) was replenished twice weekly. After 4–6 weeks, mast cells were harvested and their purity (>98%) was confirmed via flow cytometry using APC-conjugated anti-CD117 (clone 2B8; #15-1171-82; Invitrogen; 1:100 dilution, 30 minutes at 4°C).

#### Macrophages

Bone marrow-derived macrophages were cultured from 8 to 12-week-old mice in macrophage medium (24) (DMEM high-glucose medium supplemented with 10% fetal bovine serum, 100 U/mL penicillin/streptomycin, 4 mM, and 20 ng/mL macrophage colony-stimulating factor [GM-CSF, #HY-P7069, MCE]) for 7 days. Adherent cells were collected, and purity (>90%) was confirmed via flow cytometry using TLR4 antibody (#12-9924-81, eBioscience) and F4/80 antibody (#123108, Biolegend).

### Neutrophil chemotaxis: *in vitro* and *in vivo*

#### *In vitro* chemotaxis (transwell assay)

A 24-well transwell system was used with inserts precoated with 40 µL Biozellen 3D Matrigel (#B-P-00002-2; Biozellen) and stored at 4°C for up to 24 hours.

Groups: six experimental groups were created based on the contents of the lower chamber: Blank (RPMI1640 only), MC (control), VC25μM, VC250μM, MC + VC25 µM, and MC + VC250 µM. For the VC vs dehydroascorbic acid (DHA) comparison, groups included Blank, MC, VC (250 µM), DHA (250 µM), MC + VC, and MC + DHA.

Protocol: the treatment groups involved co-culturing 1 × 10⁶ mast cells with VC or DHA for 1 hour, followed by centrifugation and collection of 300 µL supernatants for the basal chamber. Neutrophils (7 × 10⁴ cells/well) were placed in the matrigel-coated upper chambers. The setup was then transferred to the JuLi Stage system (NanoEnTek Inc., Korea) for real-time analysis. Three hours after co-culture, cell migration was assessed by imaging and analyzing lower chamber infiltrates using ImageJ.

#### *In vivo* neutrophil infiltration

Corneal neutrophils were analyzed via flow cytometry: four corneas per sample were digested with 1 mg/mL collagenase I (#C0130, Sigma) at 37°C for 1 hour, filtered, and stained with antibody cocktails containing FVS510 (#564406, BD), CD45-APC (clone 30-F11; #559864, BD), CD11b-PE (clone M1/70; #50-0112-U100, TONBO), ly6G/6C-FITC (clone RB6-8C5; #11-5931-86, Invitrogen), and F4/80-Pe-Cy5.5 (clone BM8; #45-4801-82, Invitrogen) at 3 µL antibody/100 µL cell suspension (20 minutes, room temperature, light-protected). After two PBS (0.01 M, pH 7.4) washes, cells were resuspended in 500 µL PBS and analyzed using a BD FACS Canto plus flow cytometer with BD FACSDiva software (three biological replicates).

### ROS detection and NETs measuring

#### Intracellular ROS

ROS levels were measured using the Reactive Oxygen Species Assay Kit (#S0033M; Beyotime Biotechnology, Shanghai, China) via fluorescence microplate reading and flow cytometric analysis (22).

Groups: neutrophil controls (N), neutrophils challenged with live *F. solani* (N + S), and those pretreated with VC (N + S + VC, 250 µM).

Protocol: neutrophils (1.0 × 10^7^ cells/mL) were incubated with 10 µM DCFH-DA for 30 minutes at 37°C with 5% CO₂. After staining, cells were washed and immediately used for real-time ROS monitoring. For microplate measurements, neutrophils (2.0 × 10⁵ cells/well) were seeded in a 96-well plate (#LTP-021-896, BIOFIL, China), and fluorescence intensity (excitation/emission: 488/525 nm) was recorded hourly for 3 hours post-stimulation at a multiplicity of infection (MOI) of 1:1 using a BioTek Synergy H1 microplate reader (Agilent Technologies, Santa Clara, CA). For flow cytometry, neutrophils (5.0 × 10^5^ cells/well) were challenged with a pathogen at an MOI of 1:2 and sampled for 15 minutes. Cells were collected, resuspended in 300 µL 0.01 M PBS, and analyzed.

#### NETs formation

NETs formation was assessed using SYTOX Green nucleic acid stain (#S7020, Thermo Fisher Scientific) as described (25). Neutrophils were divided into three groups: controls (N), those challenged with *F. solani* (N + S, MOI = 1:1), and N + S + VC pretreatment (N + S + VC, 250 µM VC, MOI = 1:1). Following 6 hour co-culture under standard conditions (37°C/5% CO₂), SYTOX Green (1 µg/mL) was added, and fluorescence (504/523 nm) was measured using a BioTek Synergy H1 microplate reader (three replicates). Concurrently, a subset of experiments was conducted to collect culture supernatants for analysis via an alternative method, the enzyme-linked immunosorbent assay (ELISA). Absorbance was measured at 450 nm in accordance with the protocol provided in the Mouse MPO-DNA ELISA Kit (catalog number ZC-56424, ZCIBIO) Experimental Protocol Manual.

### Apoptosis of neutrophils

Annexin V Alexa Fluor 488 (#V35116, Invitrogen) and PI (#13243, Invitrogen) were used for FACS analysis to detect apoptosis of neutrophils. The neutrophils (1.0 × 10⁵ cells) were co-cultured with VC (250 µM) or DHA (250 µM) in a 12-well plate for 3 hours at 37°C with 5% CO₂. The cells were then collected and washed. The two dyes were incubated for 20 minutes at room temperature without light. After two washes, cells were resuspended for FACS analysis. Percentage of Annexin V Alexa Fluor 488 positive cells in single cells was calculated.

### Quantitative neutrophil antifungal activity

#### Time-lapse imaging

Live imaging was performed using the JuLi Stage system (10× objective; NanoEnTek Inc., Korea) with 30 minutes intervals lasting for 48 hours. Heat-inactivated fungal spores were used, and spore quantification was conducted via ImageJ particle analysis. The fungicidal index was calculated as follows:


$$Fungicidal\;Index\;(\%)=\frac{N_{0}-N_{t}}{N_{0}}\times 100\%,$$

where *N_0_* = initial spore count and *N_t_* = post-treatment count at time *t*.

#### CFU assay

Experimental groups also comprised: N, N + S, N + S + VC (250 µM VC), with different MOI ratios. Neutrophil-fungus co-cultures were homogenized after 6 hours, serially diluted (1:20) in PBS, and 100 µL aliquots were plated on PDA. Colonies were counted after 36–48 hours at 27°C using ImageJ (22).

### MIC and FIC detection

Antifungal susceptibility was tested per CLSI guidelines (M27/M38). Fungal inocula were standardized to 5.0 × 10^4^ CFU/mL (*Candida* spp.) and 5.0 × 10^3^ CFU/mL (*F. solani*) in RPMI 1640 medium (#31800-014, Gibco, 10.4 g/L, pH = 7.2, supplemented with MOPS and glucose). Serial twofold dilutions of test compounds were prepared:

Vitamin C (#MB4168; Meilunbio, Dalian, China): 131.072 to 2.048 mg/mL.

Ketoconazole (#MB1132): 2,048 to 16 µg/mL.

Amphotericin B (#MB1014): 256 to 2 µg/mL.

Voriconazole (#MB1264): 512 to 4 µg/mL.

Microdilution plates were incubated at 35°C for 24 hours (*Candida* spp.) or 28°C for 48 hours (*F. solani*). MIC endpoints were defined as the lowest concentration showing ≥90% growth inhibition compared to drug-free controls, assessed by spectrophotometric measurement at 600 nm (OD₆₀₀), and the degree of medium turbidity was observed by eyes.

For FIC determination, checkerboard assays were performed using fractional inhibitory concentrations of drug combinations. FIC was based on the checkerboard dilution method. FIC index was also calculated as follows:


$$FIC\;Index\;=\;\frac{MIC_{com,A}}{MIC_{alone,A}}+\frac{MIC_{com,B}}{MIC_{alone,B}},$$

where A and B represent paired antimicrobial agents. Synergy was defined as FIC ≤0.5, additivity 0.5–1.0, indifference 1.0–4.0, and antagonism >4.0. MIC_alone_: minimum inhibitory concentration alone; MIC_com_: minimum inhibitory concentration in combination.

All experiments included three independent biological replicates with technical duplicates.

### Reverse transcription polymerase chain reaction

To determine the expression of infection-associated cytokines in macrophages, we conducted reverse transcription polymerase chain reaction (RT-PCR). Macrophages with 1,000,000 cells/well were seeded on a 24-well plate and divided into three groups: macrophage (Mac) group, macrophage + spore (Mac + S) group, and macrophage + spore + VC (Mac + S + VC) group. The final concentration of VC was 250 µM. Cells were collected and RNA was extracted (#R6831-02, OMEGA). Then, cDNA was synthesized (#RR047A, TaKaRa), and RT-PCR was performed using QuantiNova SYBR Green PCR Kit (#208056, QIAGEN) based on GAPDH expression. The primer pairs used are listed in Table 1.

**TABLE 1:** Primers used for RT-qPCR

Gene	Forward primer	Reverse primer
IL-1β	GCAACTGTTCCTGAACTCAACT	ATCTTTTGGGGTCCGTCAACT
TNF-α	AAAATTCGAGTGACAAGCCT	CTTTGAGATCCATGCCGTTG
TGF-β	TCGACATGGATCAGTTTATGCG	CCCTGGTACTGTTGTAGATGGA
IL-10	GCTCTTACTGACTGGCATGAG	CGCAGCTCTAGGAGCATGTG
CASP-1	ACAAGGCACGGGACCTATG	TCCCAGTCAGTCCTGGAAATG
HIF-1α	GTCCCAGCTACGAAGTTACAGC	GTCCCAGCTACGAAGTTACAGC
GAPDH	AGGTCGGTGTGAACGGATTTG	TGTAGACCATGTAGTTGAGGTCA

### Graphical and statistical analysis

Flow cytometric data were analyzed using BD FACSDiva software (v8.0) or FlowJo (v10.8.1; FlowJo LLC). Statistical computations and graphical visualizations were performed in GraphPad Prism (version 9.0; GraphPad Software, San Diego, CA) employing appropriate statistical tests. A *P* value of less than 0.05 was considered to indicate statistical significance.

## RESULTS

### Protective effect of VC in fungal keratitis

**Fig 1:**
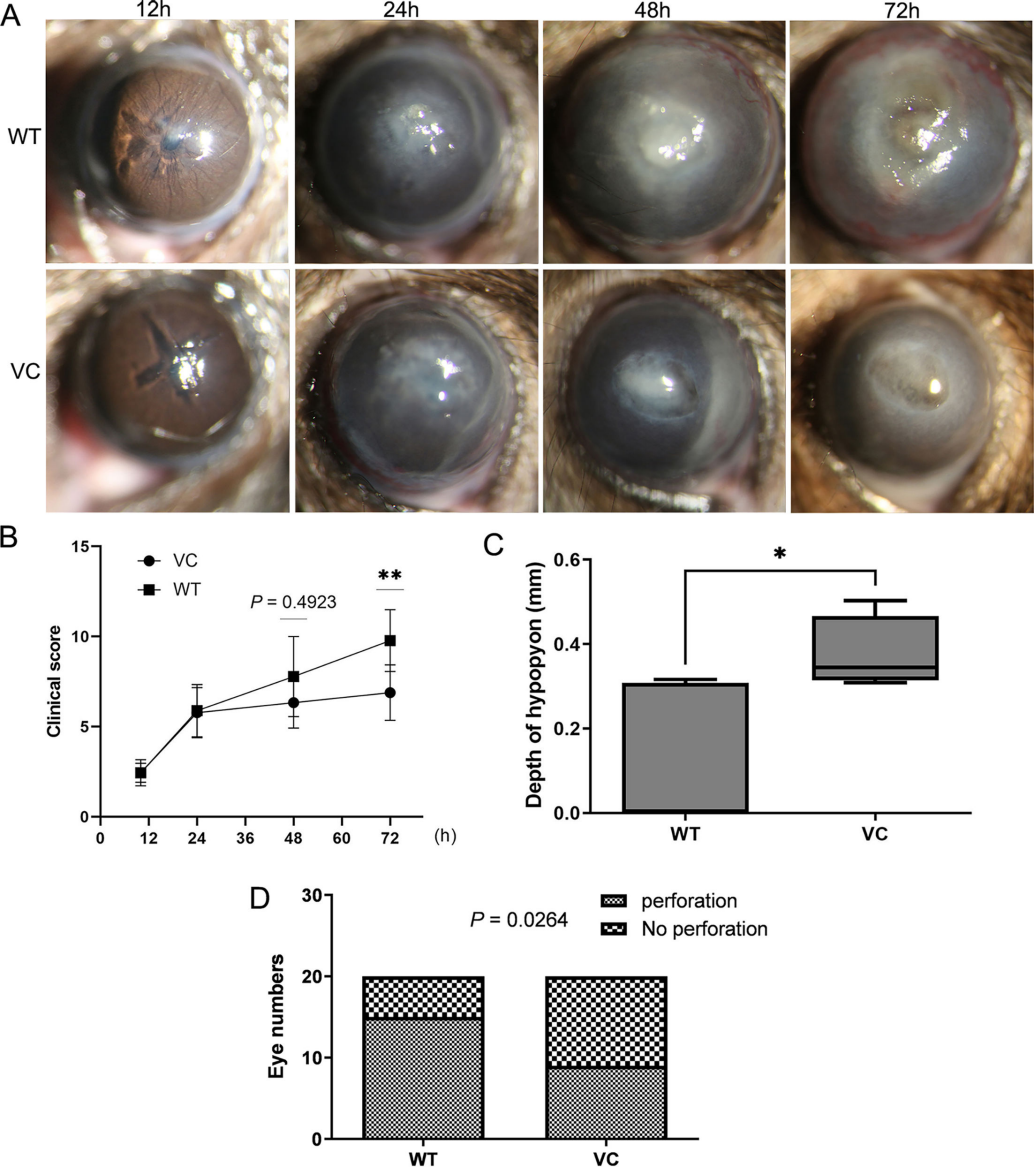
Protective effect of VC in fungal keratitis. (A) A murine model of FK induced by *F. solani* (3.1791) was established, and corneal images were captured using slip-lamp microscopy at 12, 24, 48, and 72 hours with or without VC (*n* ≥ 6 per group). (**B**) Clinical scores were compared between the WT and VC groups and showed significant differences between groups at 72 hours (*n* ≥ 6 per group). (**C**) The depth of hypopyon was measured at 24 hours, and VC treatment significantly upregulated the depth of hypopyon. (**D**) Corneal perforation rates were calculated, which showed a lower rate induced by VC (*n* ≥ 6 per group, *n* ≥ 3 independent experiments). Data are presented as mean ± SEM. Statistical analysis: repeated measures ANOVA was used for panel B, and the chi-square test was used for panel D. WT, the wild type; VC, vitamin C. *P* values are indicated as follows: *P < 0.05,**P < 0.01.

We assessed the effect of VC on FK using mice divided into two groups: the control (WT) group and the VC-supplemented (VC) group. Cornea examination was conducted at 12, 24, 48, and 72 hours post-FK induction (Fig. 1A). The WT group demonstrated hypopyon at 24 hours, ocular swelling at 48 hours, and a high clinical score at 72 hours (Fig. 1A and B). Conversely, the VC-treated group exhibited a significantly greater hypopyon than the WT group at 24 hours (*t* = 2.683, *P* = 0.031) (Fig. 1C), but a lower clinical score at 72 hours (*P* = 0.007) (Fig. 1A and B). Furthermore, corneal perforation incidence was significantly lower in the VC group, with a 45% at 72 hours compared with the WT group (*χ^2^* = 3.750, *P* = 0.026) (Fig. 1D).

### VC-induced neutrophil infiltration via mast cells

Our preliminary findings indicated an increase in hypopyon in FK induced by VC (Fig. 1C), but the specific mechanism by which VC influences neutrophil chemotaxis remains unclear. Previous research highlights the importance of mast cells in this process (21, 26). We explored whether VC could stimulate neutrophil chemotaxis via mast cells both *in vitro* and *in vivo*.

**Fig 2:**
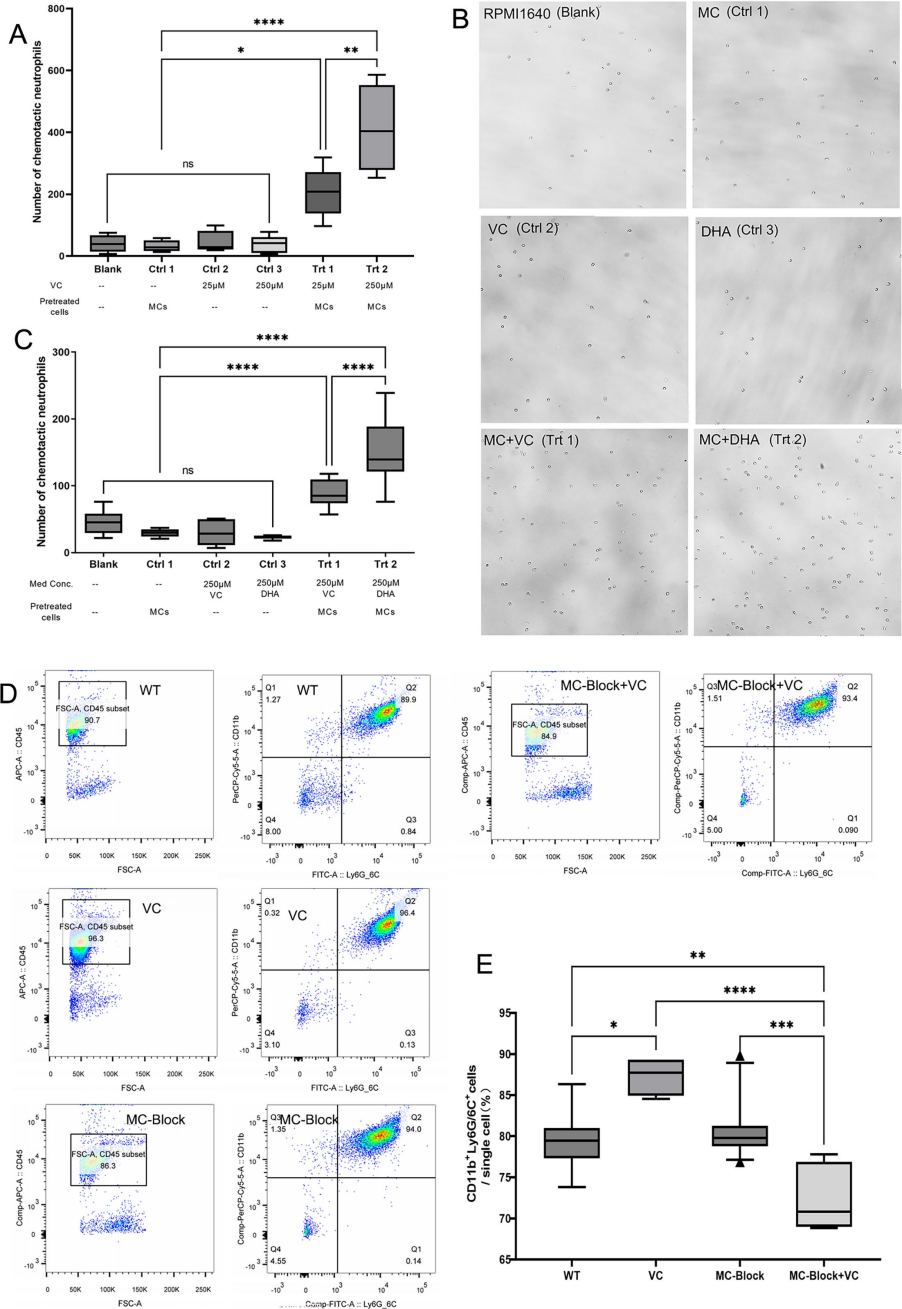
Role of mast cells in VC-induced neutrophil chemotaxis. (A) *In vitro* chemotaxis assay showing the effect of VC on MC supernatants. Neutrophil chemotactic activity was measured after 3 hours of VC exposure at 25 and 250 µM concentrations (*n* = 3 independent experiments). A concentration-dependent increase in chemotactic activity was observed. (**B, C**) Supernatants from MCs stimulated with DHA and VC attracted a comparable number of neutrophils to those from VC-treated MCs (*n* = 3 independent experiments). (**D, E**) Flow cytometry analysis of neutrophil infiltration in the cornea 24 hours after FK induction (*n* = 4 corneas in one sample, and *n* = 3 per group). Neutrophils were identified as CD11b(+) Ly6G/6C(+) cells. The VC group exhibited a significant increase in neutrophil infiltration. Addition of a mast cell membrane stabilizer to VC treatment (MC-Block + VC group) reduced neutrophil infiltration. Data are presented as mean ± SEM. Statistical analysis was performed using one-way ANOVA with Tukey’s post hoc test for panels A, B, and E. S, spore of *F. solani* (3.1791); N, neutrophils; MC, mast cell; DHA, dehydroascorbic acid, an oxidized form of vitamin; Ctrl, control; Trt, treatment; Med. Conc., medicine concentration; MC-Block, mast cell membrane stabilizer-treated groups. *P* values are indicated as follows: *P < 0.05, **P < 0.01, ***P < 0.001, ****P < 0.0001; ns indicates no significant difference.

In our *in vitro* experiment, six groups were induced as described in the part of Materials and Methods. The Blank group and the MC group without VC showed minimal neutrophil infiltration (*P* = 0.474). Cultures with various VC concentrations without MC co-stimulation had similar attraction levels to the Blank and VC groups (all *P* > 0.05) (Fig. 2A). After 3 hours of VC exposure, MC culture supernatants at 25 and 250 µM VC showed a significant concentration-dependent increase in chemotactic activity (*P* < 0.05) (Fig. 2A). DHA, the oxidized form of VC (27), also demonstrated a comparable chemotactic effect (Fig. 2B and C).

To evaluate the effect of VC on neutrophil infiltration in the cornea, flow cytometry was employed 24 hours after FK model induction. In the water-treated control group (WT group), neutrophils made up 79.60% ± 3.80% of the single cell population (Fig. 2D and E). The VC group exhibited a significant increase to 87.32% ± 2.37% (*P* = 0.010). The mast cell membrane stabilizer alone (MC-Block) did not significantly differ from the WT group (*P* = 0.8736), but the combination with VC (MC-Block + VC group) reduced neutrophils to 72.32% ± 3.89%, significantly lower than the VC (*P* < 0.001), the WT group (*P* = 0.007), and the MC-Block groups (*P* < 0.001).

### VC-mediated enhancement of neutrophil fungicidal activity: quantity over ROS and NETs

In light of the role and mechanisms of neutrophils in fungicidal activity, particularly through the formation of ROS and NETs, we investigated the potential influence of VC on these processes.

**Fig 3:**
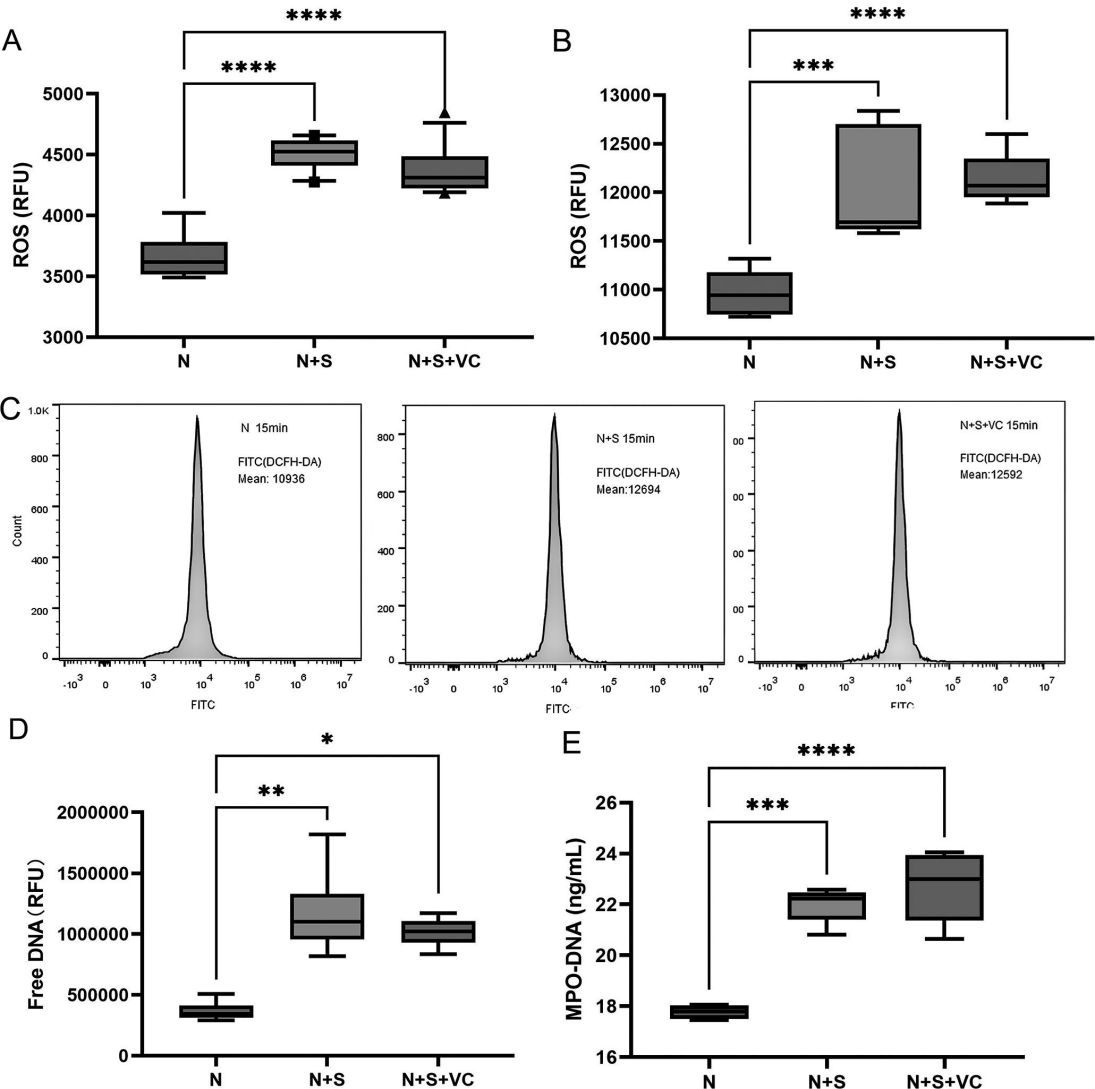
Effect of VC on ROS generation and NET formation. (A) Measurement of ROS production in neutrophils using a fluorescence assay (*n* = 8 per group and *n* = 3 independent experiments). VC supplementation (N + S + VC) did not significantly reduce ROS production compared to N + S. (**B, C**) Flow cytometry analysis of ROS production in neutrophils (*n* = 3 per group and *n* = 3 independent experiments). Neutrophils exhibited low baseline ROS levels (N group). VC treatment did not significantly reduce ROS levels compared with resting neutrophils. (**D, E**) Evaluation of NET formation was conducted using SYTOX Green staining in a fluorescence assay and MPO-DNA ELISA at 450 nm absorption after 6 hours (*n* = 6 per group and *n* = 3 independent experiments). Fungal spores induced significant NET formation at 6 hours; VC treatment did not significantly reduce NET formation compared with untreated controls. Data are presented as mean ± SEM. Statistical analysis was performed using one-way ANOVA with Tukey’s post hoc test for panels A, C, and D. N, neutrophils; S, spore of *F. solani* (3.1791); DHA, dehydroascorbic acid, an oxidized form of vitamin; RFU, relative fluorescence unit. *P* values are indicated as follows: *P < 0.05, **P < 0.01, ***P < 0.001, ****P < 0.0001; ns indicates no significant difference.

Previous studies show VC influences ROS production in neutrophils (28, 29). Given the differences between fungi and other microbes, we explored VC’s impact on ROS during neutrophil-driven fungal killing. Our results showed that fungal spores significantly increased ROS in neutrophils, but VC did not notably reduce this increase (Fig. 3A). Flow cytometry confirmed that VC does not suppress ROS production in this context (Fig. 3B and C). Initially, neutrophils had low ROS levels, which rose significantly upon exposure to fungi at 15 minutes (*P* < 0.001). VC did not significantly decrease ROS, which remained higher than in resting neutrophils (*P* > 0.05) (Fig. 3B and C). This suggests VC may not impact ROS production during neutrophil-driven fungal spore killing.

SYTOX Green staining and MPO-DNA ELISA revealed that spores induced NET formation during neutrophil-mediated spore eradication, with a notable increase at 6 hours (Fig. 3D and E ). The addition of VC did not significantly decrease NET formation (*P* = 0.264 for Fig. 3D, and *P* = 0.481 for Fig. 3E, N + S vs N + S + VC).

**Fig 4:**
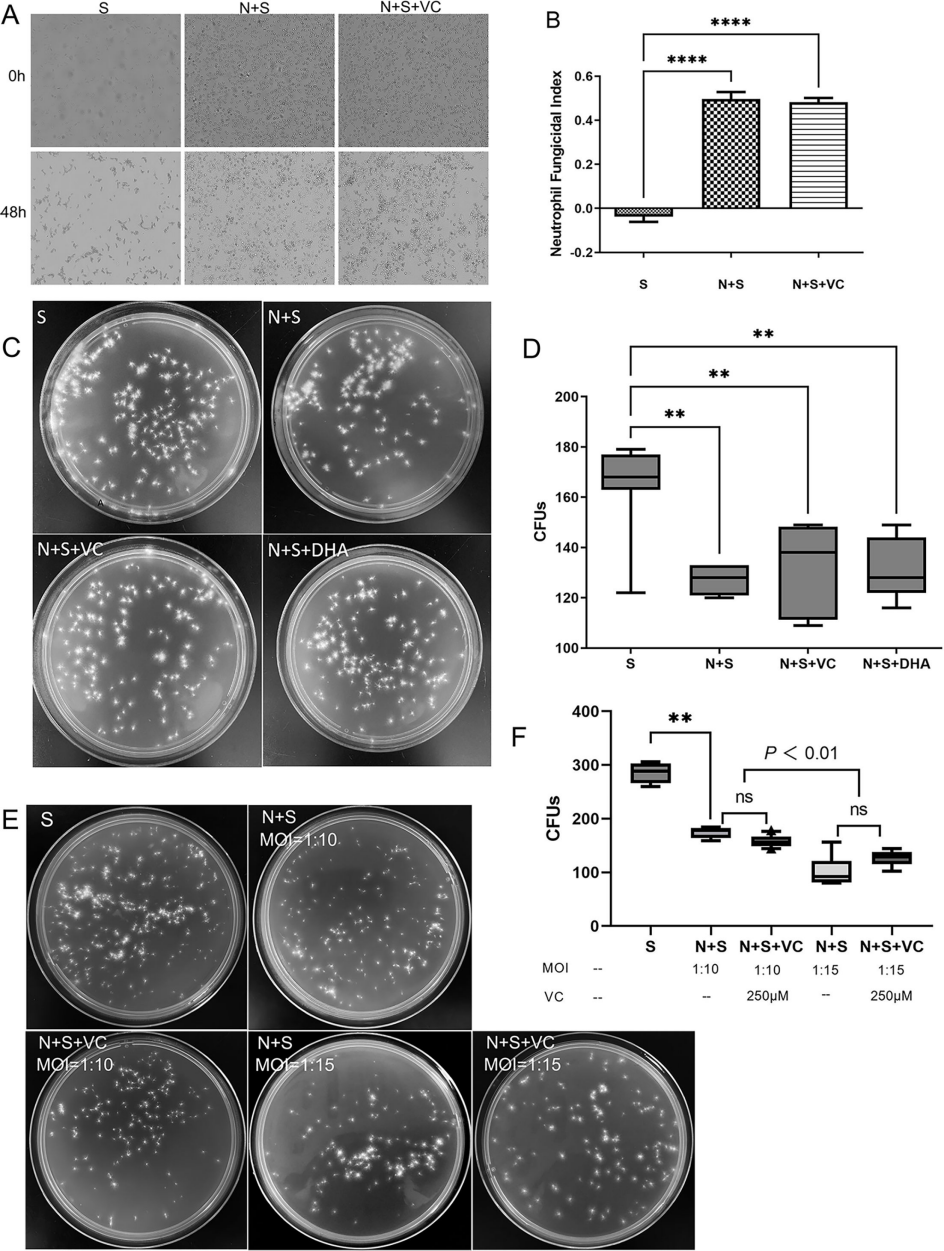
VC-induced elevated quantity of neutrophils promoted fungicidal activity. (**A, B**) Real-time observational approach to assess the fungicidal index after a 48 hour co-incubation of neutrophils, fungal spores, and VC (10× objective lens) (*n* = 4 per group and *n* = 3 independent experiments). Fungicidal indices were calculated, and there was no significant difference between the VC-treated and control groups. (**C, D**) CFU counting to evaluate the fungicidal activity of neutrophils after a 6 hour co-culture with spores and VC (*n* = 6 per group, *n* = 3 independent experiments). The addition of VC or DHA did not further reduce CFU counts compared with the neutrophil-added (N + S) group, although CFU counts remained lower than those in the S group. (**E and F**) *In vitro* experiments using different MOI ratios to investigate the role of neutrophil quantity in fungal killing. At an MOI of 1:10, VC did not significantly reduce CFUs. At an increased MOI of 1:15, VC did enhance fungal killing compared with an MOI of 1:10. Data are presented as mean ± SEM. Statistical analysis was performed using one-way ANOVA with Tukey’s post hoc test for panels A, B, C, and D. N, neutrophils; DHA, dehydroascorbic acid, an oxidized form of vitamin; *P < 0.05, **P < 0.01, ***P < 0.001, ****P < 0.0001; ns indicates no significant difference.

To assess VC’s impact on neutrophils' ability to eliminate fungal spores, a real-time observation method and fungicidal index were used. After 48 hours, the fungicidal index in the VC-treated (N + S + VC) group was not significantly different from the control group with only neutrophils and spores (N + S) (*P* = 0.923) (Fig. 4A and B).

The impact of VC on the fungicidal activity of neutrophils was further assessed through CFU enumeration. In the experiment, VC was cultured with fungal spores and neutrophils for six hours. Spores alone (S group) showed high CFUs, indicating basal growth capacity. Neutrophils (N + S group) significantly reduced fungal growth (*P* < 0.01), demonstrating their fungicidal ability. Adding VC (N + S + VC group) or DHA (N + S + DHA group) did not further decrease CFUs compared to the N + S group (*P* > 0.05), but CFUs were still significantly lower than in the S group (all *P* < 0.01) (Fig. 4C and D).

Based on the outcomes from *in vivo* experiments examining neutrophil infiltration and their protective function in FK, we propose that VC augments the fungicidal activity of neutrophils by increasing their population. To further elucidate the role of neutrophil quantity in fungal eradication, experiments *in vitro* were conducted utilizing various MOI ratios (Fig. 4E and F). At an MOI of 1:10, neutrophils significantly reduced CFUs compared with the control group containing spores alone (*P* = 0.0012, vs S group), and VC did not significantly reduce CFUs (*P* = 0.2447, vs N + S group, MOI = 1:10). When the MOI was increased to be 1:15 (with an additional 50% neutrophils), CFUs were further reduced (*P* = 0.0016, vs N + S group, MOI = 1:10). Additionally, although VC did not significantly affect the ability of neutrophils to kill fungi at an MOI of 1:15 (*P* = 0.2265, vs N + S group, MOI = 1:15), it did enhance fungal killing compared to the MOI of 1:10 (*P* = 0.0016, vs N + S group, MOI = 1:10).

The underlying mechanism driving increased neutrophil numbers (e.g., enhanced infiltration, prolonged survival, or local proliferation) remains to be determined in future studies, as the current data do not distinguish these possibilities.

These findings indicate that VC does not alter the production of ROS or neutrophil extracellular traps (NETs). Instead, there may be an association between neutrophil accumulation and improved fungal clearance following VC treatment, and the related regulatory pathways warrant further investigation.

### Modulated macrophage immune response and elevated neutrophil apoptosis induced by vitamin C

Based on the experimental results, VC was found to reduce the incidence of corneal perforation and downregulate clinical scores. We hypothesized that these outcomes may result from VC’s regulation of macrophage responses and induction of neutrophil apoptosis.

**Fig 5:**
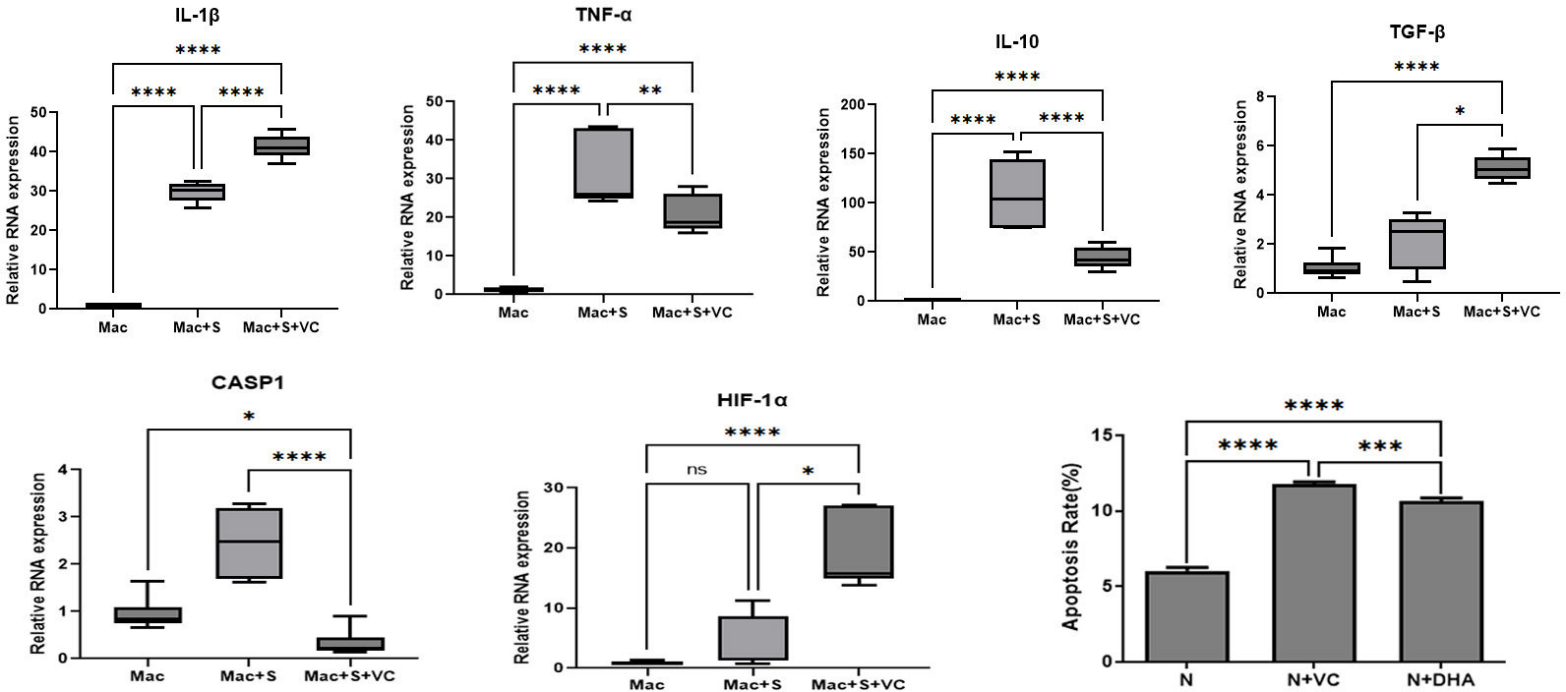
Modulated macrophage immune response and elevated neutrophil apoptosis induced by VC. Bone marrow-derived macrophages were co-cultured with spores for 3 hours, with or without VC, and the relative mRNA levels of IL-1β, TNF-α, IL-10, TGF-β, CASP-1, and HIF-1α were assessed by RT-PCR. Neutrophil apoptosis was evaluated via FACS analysis after 3 hours of VC or DHA exposure (bottom right). Mac, bone marrow-derived macrophage; N, neutrophils; DHA, dehydroascorbic acid, oxidized form of vitamin. *P* values are indicated as follows: *P < 0.05, **P < 0.01, ***P < 0.001, ****P < 0.0001, ns: no significant difference.

We examined the impact of VC on the mRNA expression of inflammatory factors in bone marrow-derived macrophage cells following fungal stimulation, utilizing RT-PCR. Quantitative RT-PCR analysis demonstrated that exposure to spores alone (Mac + S group) resulted in elevated mRNA levels of IL-1β, TNF-α, and IL-10, while the mRNA levels of HIF-1α and TGF-β remained unchanged (Fig. 5). The introduction of VC (Mac + S + VC group) modified this expression pattern: there was a significant reduction in the mRNA expression of TNF-α and IL-10 compared to the Mac + S group (all *P* < 0.01), whereas the mRNA levels of IL-1β, HIF-1α, and TGF-β were significantly increased (all *P* < 0.05). Despite the increased IL-1β mRNA levels, the expression of caspase-1 (CASP1) mRNA (Fig. 5), which is crucial for IL-1β maturation, was significantly reduced, ultimately potentially mitigating the pro-inflammatory function of IL-1β. These findings indicate that VC may exert anti-inflammatory effects by suppressing CASP1-mediated IL-1β maturation (as indicated by downregulated CASP1 mRNA expression, though this requires protein-level validation) while concurrently up-regulating HIF-1α.

We subsequently investigated whether VC also promotes neutrophil apoptosis, a necessary step for their safe clearance by macrophages during the late stages of FK. Apoptosis rates were assessed in the presence and absence of VC and DHA treatment. At the resting stage, neutrophils exhibited lower apoptosis rates. Both VC and DHA treatments significantly increased apoptosis rates (all *P* < 0.01), with VC demonstrating a more pronounced effect compared to DHA (*P* < 0.01) (Fig. 5).

Collectively, these findings indicate that VC curbs the cytokine storm within BMDM and subsequently facilitates the efficient removal of spent neutrophils, thereby transforming robust early immunity into sustained corneal protection.

### Additional antifungal effect of VC with amphotericin B in fungal keratitis

The antifungal properties of vitamin C have not been extensively investigated. This study aimed to determine the MIC of vitamin C and to explore its potential synergistic effects with established antifungal agents. Utilizing a drug susceptibility assay, we assessed the MIC across four fungal species. Notably, the MIC for vitamin C was consistently observed to be 65.536 mg/mL, as presented in Table 2.

**TABLE 2:** Minimum inhibitory concentration of vitamin C

Fungi strains	MIC (mg/mL)
*Fusarium solani F*.3.5840	65.536
*Fusarium solani F*.3.1791	65.536
*Candida albicans*	65.536
*Candida parapsilosis*	65.536

The drug susceptibility testing for fungi yielded significant insights into the efficacy of antifungal agents against specific pathogens. Ketoconazole demonstrated a MIC of 512 µg/mL against *Candida parapsilosis*, whereas voriconazole showed a lower MIC of 64 µg/mL against the same organism. The FIC index for ketoconazole was 1.000, suggesting no interaction, while voriconazole had a FIC index ranging from 0.516 to 1.000, indicative of an additive effect.

In assays against *F. solani* F.3.5840, AmB showed an MIC of 16 µg/mL, and voriconazole had an MIC of 64 µg/mL. The FIC indices for AmB and voriconazole were observed to range from 0.516 to 1.000 and 0.516 to 0.750, respectively (as detailed in Table 3), indicating additivity effect.

**TABLE 3:** Additive effect between vitamin C and anti-fungal reagents^*a*^

Fungi strains	Antifungal reagents	MIC_alone_ μg/mL	MIC_com_ μg/mL	FIC index
*Fusarium solani F*.3.5840	Amphotericin B	16	2–8	0.516–1.000
	Voriconazole	64	8–16	0.516–0.750
	VC	65,536	32,768	–^*b*^
*Candida parapsilosis*	Ketoconazole	512	256	1.000
	Voriconazole	64	16–32	0.516–1.000
	VC	65,536	32,768	–

^*a*^ The FIC index indicates the interaction between two substances. An index of less than 0.5 indicates synergy,0.5-1 for additive effect,1-2 for irrelevance, and greater than 2 for antagonism. MIC_alone_:Minimum Inhibitory Concentration alone; MIC_com_: Minimum Inhibitory Concentration in combination.

^*b*^ The “–” indicates not applicable.

These findings emphasize the direct fungicidal activity of VC *in vitro* and its potential to augment the efficacy of certain antifungal agents, underscoring its prospective utility in therapeutic combinations for the treatment of fungal infections.

**Fig 6:**
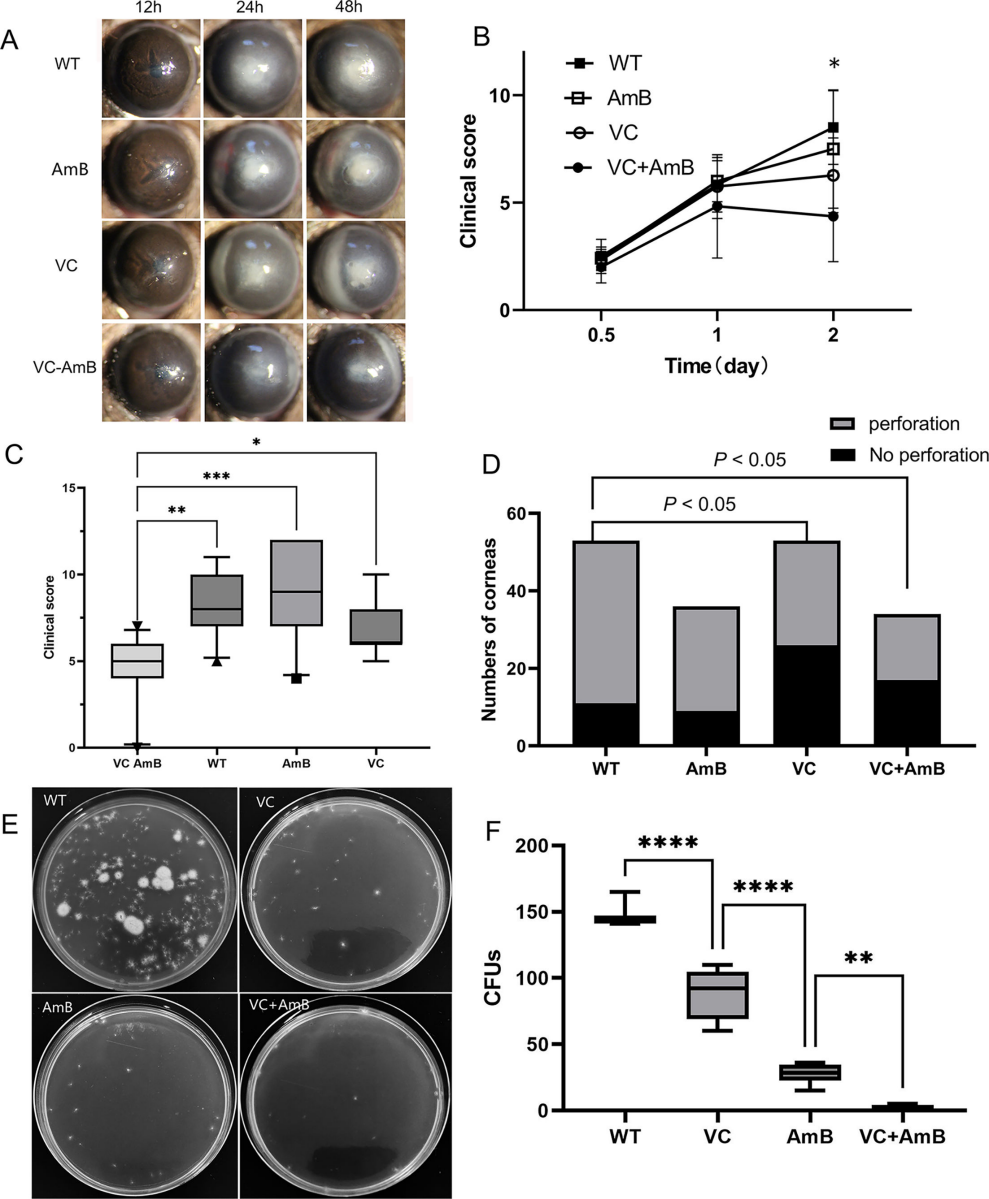
Additive effects of VC and amphotericin B in treating FK. (A) Representative slit-lamp photographs of corneal changes in murine models of FK induced by *F. solani* (3.1791) at 12, 24, and 48 hours post-treatment (*n* = 12 eyes per group). (**B**) Clinical scores were significantly different among WT, VC, and VC-AmB groups over the 48 hour observation period (repeated-measures ANOVA) (*n* = 12 eyes per group). (**C**) Both VC and VC-AmB groups showed reduced clinical scores compared to WT at 48 hours (*n* = 12 eyes per group). (**D**) The VC-AmB group had a significantly lower rate of corneal perforation compared to the WT group at 48 hours. (**E, F**) Quantitative analysis of fungal load using PDA plate cultures of corneal tissue suspensions at 72 hours post-treatment (*n* ≥ 4 corneas per group). The VC-AmB group had significantly fewer CFUs than the WT, VC, and AmB groups, indicating an additive antifungal effect. Data are presented as mean ± SEM. Statistical analysis was performed using repeated measures ANOVA for panel B, one-way ANOVA with Tukey’s post hoc test for panels C and E, and chi-square test for panel D. FK, fungal keratitis; WT, the wild type; VC, vitamin C; AmB, amphotericin B; CFU, colony-forming unit. *P* values are indicated as follows: *P < 0.05, **P < 0.01, ***P < 0.001, ****P < 0.0001.

To evaluate the potential additive effects of combining VC with antifungal agents in the treatment of FK, a series of *in vivo* experiments were conducted. The study involved a comparative analysis of clinical scores and corneal perforation rates across four groups: wild type (WT) as the control, VC alone, AmB treated, and VC combined with AmB (VC + AmB). Significant differences were observed among these groups for both clinical scores (two-way ANOVA, *F* = 4.920, *P* = 0.005) and perforation rate (chi-square test, *χ^2^* = 14.08, *P* = 0.003) (Fig. 6A through D).

At the 48 hour mark, the WT group exhibited elevated clinical scores and a high perforation rate (Fig. 6A through D). Conversely, the VC group demonstrated significantly reduced clinical scores (*P* = 0.0492) and perforation rate (*P* < 0.05) relative to the WT group (Fig. 6C and D). Although AmB displayed a low MIC against *F. solani* strain 3.5840, the AmB monotherapy group showed clinical scores and perforation rates that were indistinguishable from those of the WT group (all *P* > 0.05). However, the VC + AmB group showed lower clinical scores (*P* < 0.001) and perforation rates (*P* < 0.05) compared to the WT group at 48 hours. Notably, even the VC group, which did not receive AmB, exhibited a significantly lower corneal perforation rate compared to the WT group (*P* < 0.05). Although the VC + AmB group did not achieve statistical separation from the VC group for either clinical score (*P* = 0.462) or perforation rate (*P* > 0.05), the AmB antifungal activity favored the combination, suggesting an additive rather than synergistic interaction.

The enhanced antifungal effect when VC was combined with AmB in the treatment of FK was further investigated by reduced fungal load in the cornea at 72 hours among four groups: WT, VC, AmB, and VC + AmB (Fig. 6E and F). Corneal tissue suspensions were collected at 72 hours and cultured on PDA plates for quantitative CFU analysis. The results indicated that the VC group exhibited a significantly lower number of CFUs compared to the WT group (*P* < 0.001). The AmB monotherapy group showed a lower number of CFUs than the WT and VC groups. Importantly, the VC + AmB group exhibited a lower CFU than the VC group (*P* = 0.004) and the AmB group (*P* < 0.001). These findings underscore the additive antifungal effect of combining VC with AmB in reducing the corneal fungal load.

## DISCUSSION

Fungal keratitis poses a major global threat to ocular health, with limited effective antifungals, rapid fungal proliferation, and a high risk of irreversible blindness or invasive surgical intervention (1). This unmet clinical need underscores the urgency of identifying safe, accessible adjunctive therapies—and our study in a mouse FK model identifies VC as a promising candidate by delineating its multifaceted role in enhancing antifungal immunity while mitigating tissue damage.

VC significantly lowers corneal perforation rates in a murine model of FK, though its mechanisms remain incompletely understood. Guinea pigs with congenital VC deficiency exhibit heightened susceptibility to infections, underscoring VC’s potential role in immune defense (2). Similarly, VC supplementation has shown significant adjunctive benefits in sepsis, reducing mortality and enhancing neutrophil function (30–32). Our study demonstrates that VC, when administered via drinking water, reduces corneal perforation rates and preserves ocular integrity in severe FK.

Neutrophil chemotaxis—a critical step in microbial clearance—is enhanced by VC. Our experiments confirm that VC promotes neutrophil infiltration in FK, both *in vitro* and *in vivo*, aligning with prior studies. VC deficiency impairs neutrophil migration to infection sites (29), while dietary VC supplementation improves chemotaxis and increases neutrophil counts in sepsis patients (33). VC may indirectly enhance chemotaxis by detoxifying histamine, independent of fMLP or IL-8 receptors (34, 35).

Mast cells may mediate VC’s effect on chemotaxis (11). Others and our previously published data have shown that VC influences mast cell-driven neutrophil migration, though evidence is conflicting (11, 21, 35, 36). Our *in vitro* and *in vivo* experiments confirmed that VC affected mast cell-mediated neutrophil migration. Opposite evidence suggests that 2 mM vitamin C could reduce mast cell degranulation, but at low concentrations, vitamin C does not affect degranulation (36). Recent studies have shown that a variety of drugs can cause non-classical activation of mast cells (11, 37–39), and it is speculated that VC may have a similar effect. Whether VC induces neutrophil chemotaxis through any atypical activation of mast cells still requires further investigation.

Neutrophils play a critical role in immune defense by ROS generation and NET formation. VC exhibits a dual role in it. Low concentrations of VC (<1 mM) are known to inhibit ROS generation and NET formation, whereas high concentrations (>10 mM) promote it (8, 9, 29, 30, 40–42). However, in our study, low-dose VC (250 µM) did not appear to influence ROS production in mouse bone marrow-isolated neutrophils, possibly due to variations in the experimental microenvironment. These findings highlight the complex VC effects on neutrophils and underscore the need for further investigation to clarify its thresholds and microorganism-specific effects.

Despite VC’s lack of direct impact on ROS or NETs, our *in vitro* experiments confirmed that VC enhances neutrophil-mediated fungal clearance by increasing neutrophil numbers, rather than modulating ROS or NETs. The underlying mechanism driving increased neutrophil numbers (e.g., enhanced infiltration, prolonged survival, or local proliferation) remains to be determined in future studies.

Our findings delineate a previously unrecognized mechanism by which VC reconciles robust neutrophil recruitment with protection against corneal perforation in experimental FK. By simultaneously restraining macrophage-derived TNF-α and accelerating neutrophil apoptosis, VC redirects a potentially destructive inflammatory cascade toward controlled resolution. Although VC raised IL-1β mRNA, it simultaneously suppressed CASP1, the inflammasome effector required for IL-1β processing (4). This dissociation between transcription and bioactivity aligns with recent observations that CASP1 inhibition can neutralize IL-1β-mediated tissue injury without altering its gene expression (43). The observed CASP1 mRNA downregulation provides preliminary evidence for VC’s potential regulatory role in the CASP1-IL-1β axis, while emphasizing it as a hypothesis to be further tested with protein-level assays. The parallel induction of HIF-1α is also mechanistically congruent: HIF-1α not only restrains excessive inflammasome activation under hypoxic conditions but also promotes TGF-β-dependent tissue repair (44, 45). Thus, VC appears to recalibrate the IL-1β signaling network from pro-inflammation to repair.

Neutrophil persistence is a well-established driver of corneal damage (46). Here, VC augmented apoptosis, consistent with findings in human peripheral blood neutrophils (16), and its oxidized form, DHA, also contributed to this effect. Apoptotic neutrophils provide “find-me” signals that license macrophages to perform non-phlogistic phagocytosis, a process shown to reduce bystander damage in sterile inflammation models (47). Our data extend this paradigm to an infectious setting, suggesting that VC couples the termination of neutrophil life span to macrophage-mediated clearance.

VC also demonstrated additional antifungal activity with AmB. Some studies have found that VC alone does not inhibit the growth of *Candida albicans,* but others showed a direct inhibitory effect on *Aspergillus parasiticus* (48). The capacity of VC to enhance the fungicidal efficacy of amphotericin B against *Candida albicans* and *Cryptococcus neoformans* has been noted (49), potentially through the facilitation of VC-induced H_2_O_2_ influx via pores on the fungal cell surface created by amphotericin-mediated (50). Our study indicated an independent fungicidal effect of VC, which may be due to either concentrations differing in previous studies or susceptibilities varying among different fungal species. Our study further showed that VC augmented the efficacy of voriconazole against *Candida parapsilosis* and *F. solani*, the efficacy of amphotericin B on *F. solani F*.3.5840, and ketoconazole against *Candida parapsilosis*. The mechanism underlying the additional fungicidal effect of VC and voriconazole remains unclear and warrants further investigation. But the high MIC values limited the effect, suggesting that VC’s role may be more related to immune modulation or synergistic effects rather than direct antifungal action.

Our study revealed that mice supplemented with VC alone exhibited a lower fungal load in the cornea compared to those in the WT group at 72 hours. This observation likely stems from two key factors. First, VC significantly increased neutrophil counts, enhancing their antifungal activity independently of ROS or NETs. This suggests VC primarily augments neutrophil quantity rather than modulating their functional pathways. Second, VC possesses direct antifungal properties, as demonstrated in our study and others, by disrupting fungal metabolic pathways or bolstering host immunity. Combined with enhanced neutrophil activity, these effects likely contributed to the reduced fungal load in VC-treated mice.

The combination of VC and AmB demonstrated superior efficacy, attributed to additional mechanisms. VC enhanced neutrophil recruitment to infection sites, strengthening the immune response against fungi. Additionally, VC’s water solubility may improve AmB delivery to corneal tissue, while facilitating H₂O₂ influx via pores created by AmB on fungal cell surfaces (49). These interactions highlight the potential of VC-AmB as a potential therapeutic strategy for FK and severe fungal infections.

The route of VC administration influences its effects. While enteral administration maintains physiological levels, intravenous dosing is required for therapeutic efficacy (30). Our experiments used oral VC supplementation in mice, leveraging their small size to ensure local infections impacted systemic immunity. Topical VC formulations already demonstrate safety in ocular burn models (51). Coupled with our mechanistic evidence, these data provide a rationale for repurposing VC as an adjunctive therapy for FK, particularly in settings where systemic anti-fungal penetration is poor. Future randomized trials should evaluate whether VC eye drops can reduce the need for emergency therapeutic keratoplasty.

However, our study has several limitations: unclear mechanism of VC reducing corneal perforation, murine models not fully replicating human FK, systemic VC not replacing local ophthalmic use, and lack of protein-level validation for CASP1 (cleaved/active) and mature IL-1β (to be verified via western blot/ELISA). Additionally, only neutrophils and MCs were studied—future work should explore VC’s effects on more immune cells and its clinical use in human FK.

### Conclusions

Our findings indicate that VC can enhance neutrophil chemotaxis and modulate macrophage function, significantly reducing the risk of corneal perforation in murine models of FK. VC likely achieves this by enhancing neutrophil chemotaxis through mast cells, not NET formation or ROS production, and by modulating macrophage anti-inflammatory functions, providing additional antimicrobial effects, reducing fungal loads, and improving outcomes. Thus, VC could be an adjunct therapy to reduce corneal damage in FK.

## Data Availability

Data will be made available on request.

## SUPPLEMENTAL MATERIAL

Fig. S1 aac.01165-25-s0001.tif Mouse body weight changes following 7-day different drinking water treatment.

Fig. S2 aac.01165-25-s0002.tif Typical images of corneal perforation.

Fig. S3 aac.01165-25-s0003.tif A stepwise gating strategy to identify and quantify neutrophils in mouse corneal single-cell suspensions.

Fig. S4 aac.01165-25-s0004.tif Blank control for CFU assay.

Fig. S5 aac.01165-25-s0005.tif Representative images of MIC plates.

Supplemental material aac.01165-25-s0006.docx Supplemental figure legends.

ASM does not own the copyrights to Supplemental Material that may be linked to, or accessed through, an article. The authors have granted ASM a non-exclusive, world-wide license to publish the Supplemental Material files. Please contact the corresponding author directly for reuse.
