# Supplementary figures and images for: Vitamin C enhances corneal fungal infection treatment in mice via chemotaxis and anti-inflammation
Source: Antimicrob Agents Chemother. 2025 Nov 28;70(1):e01165-25. doi: 10.1128/aac.01165-25 (PMC12777558; doi:10.1128/aac.01165-25)

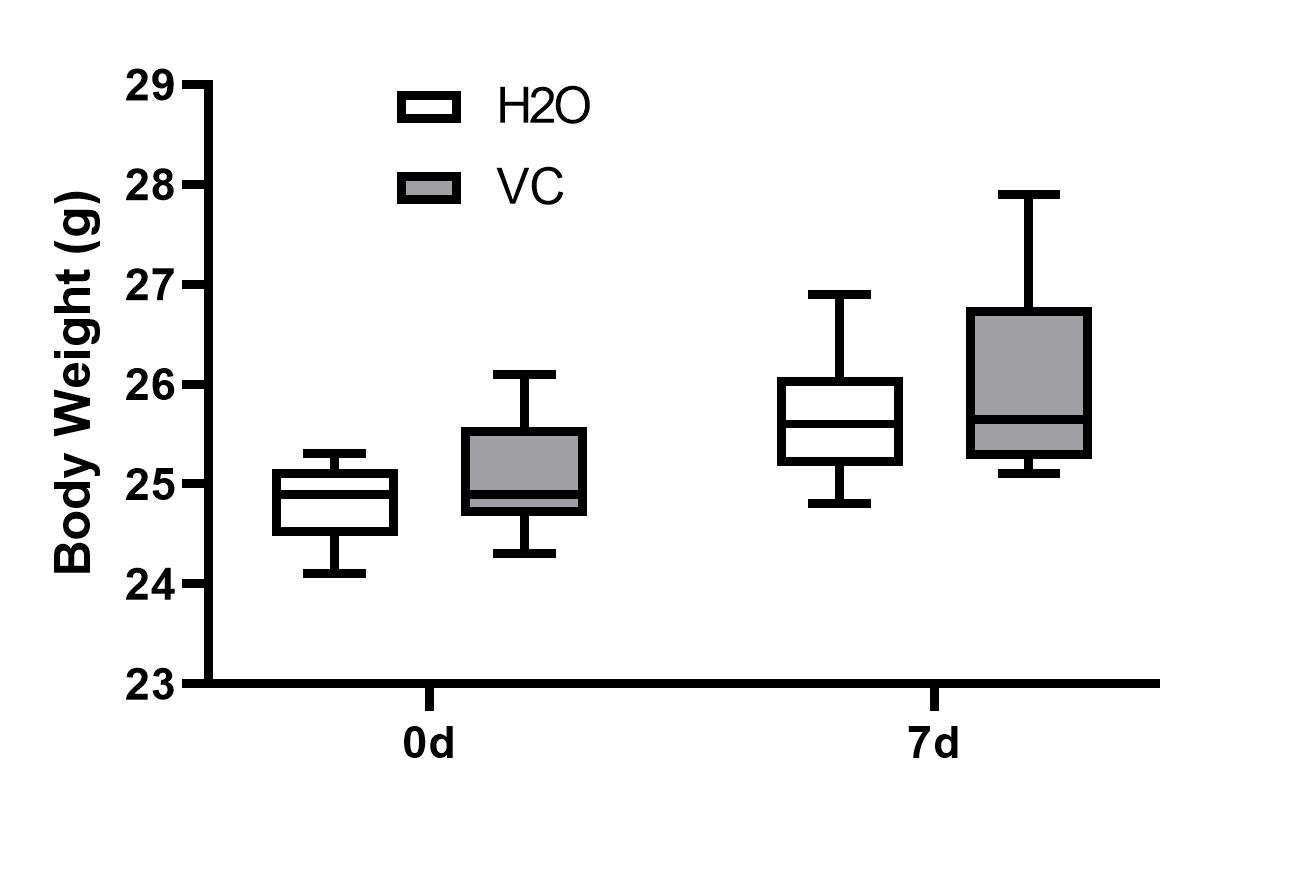

Supplement: Fig. S1 — Mouse body weight changes following 7-day different drinking water treatment. [file aac.01165-25-s0001.tif]

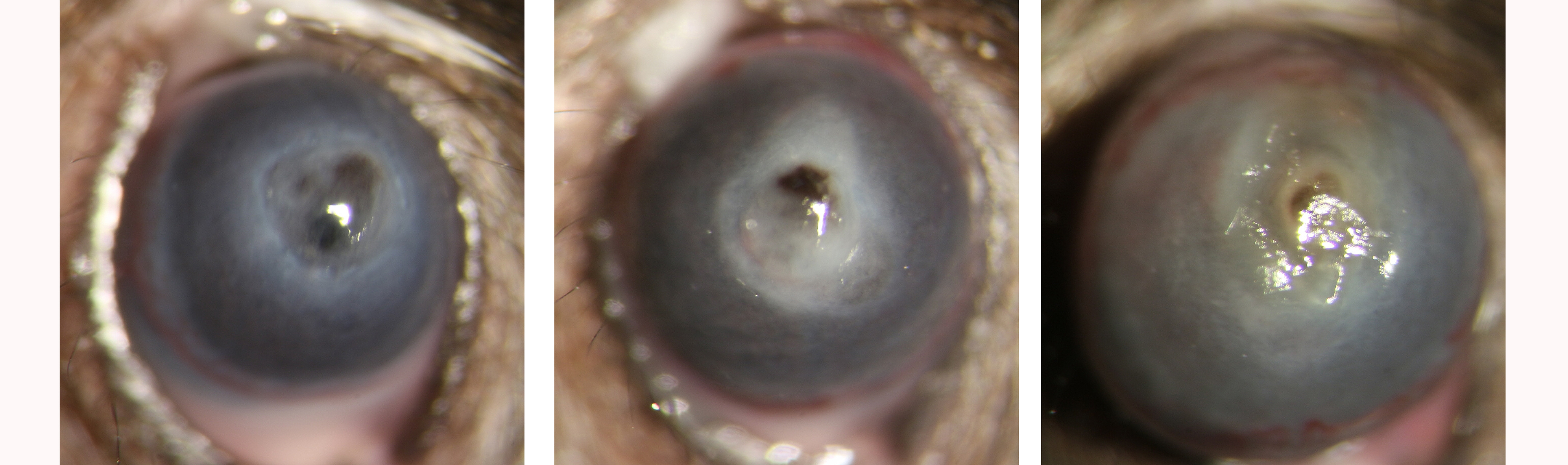

Supplement: Fig. S2 — Typical images of corneal perforation. [file aac.01165-25-s0002.tif]

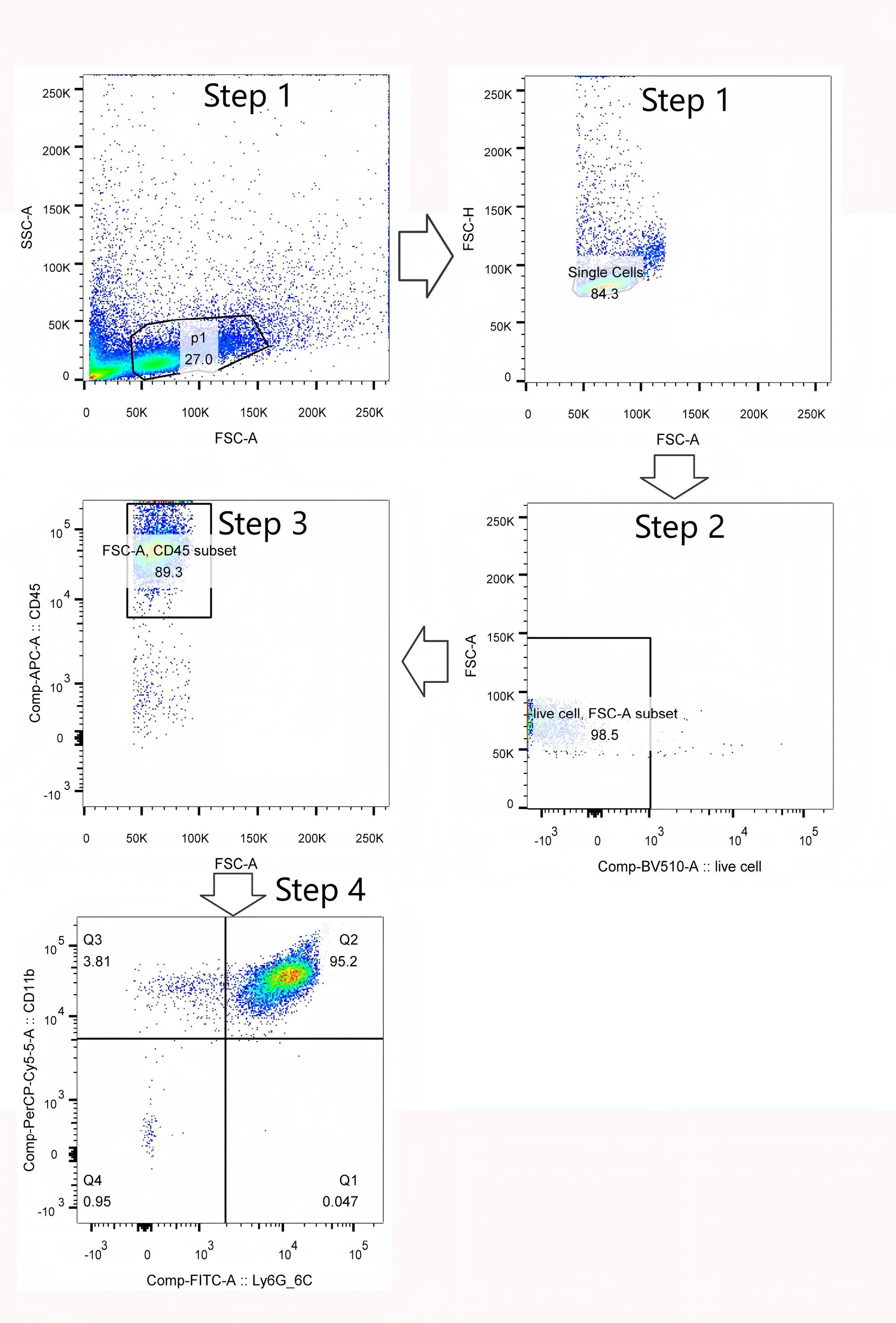

Supplement: Fig. S3 — A stepwise gating strategy to identify and quantify neutrophils in mouse corneal single-cell suspensions. [file aac.01165-25-s0003.tif]

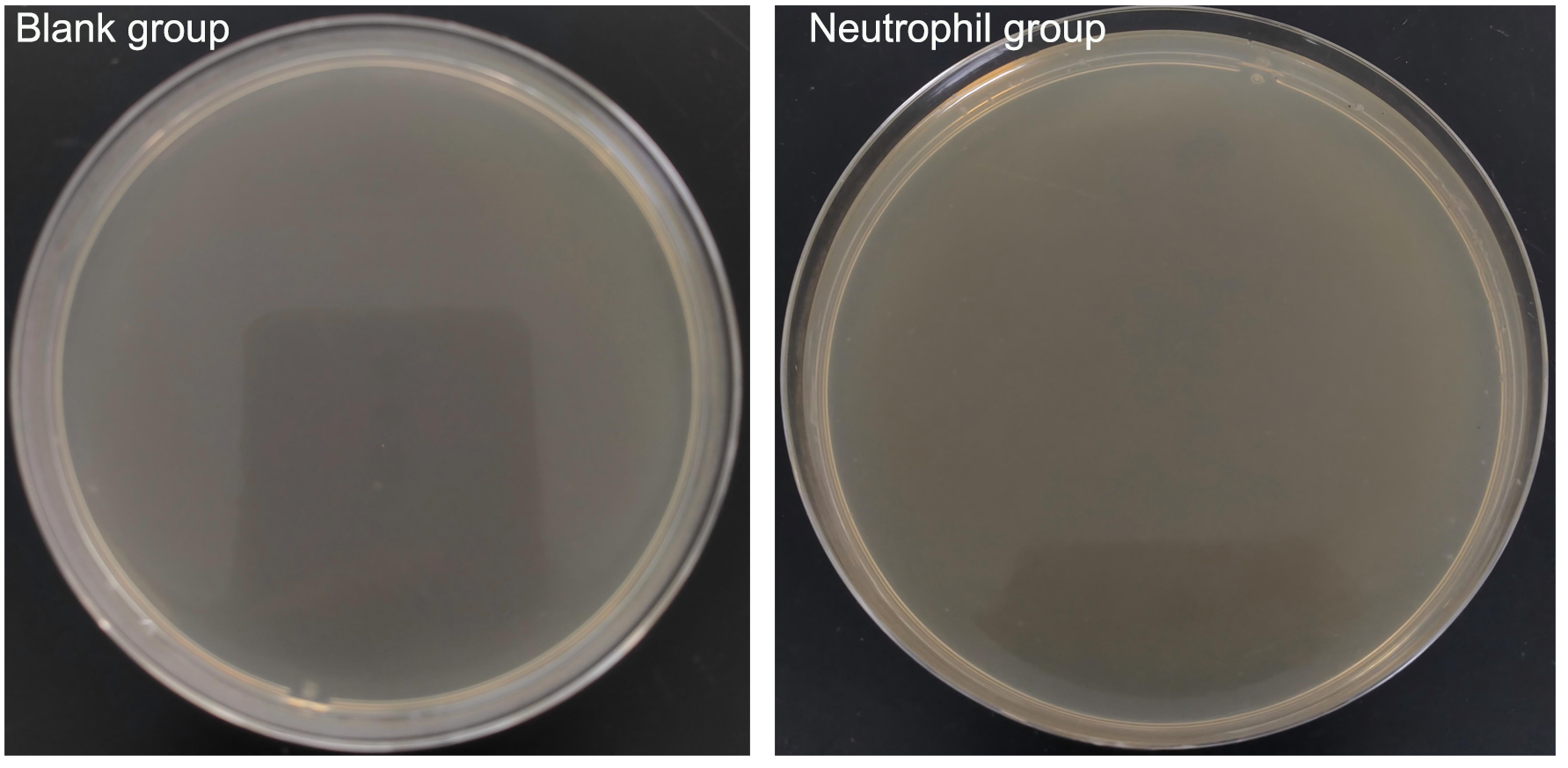

Supplement: Fig. S4 — Blank control for CFU assay. [file aac.01165-25-s0004.tif]

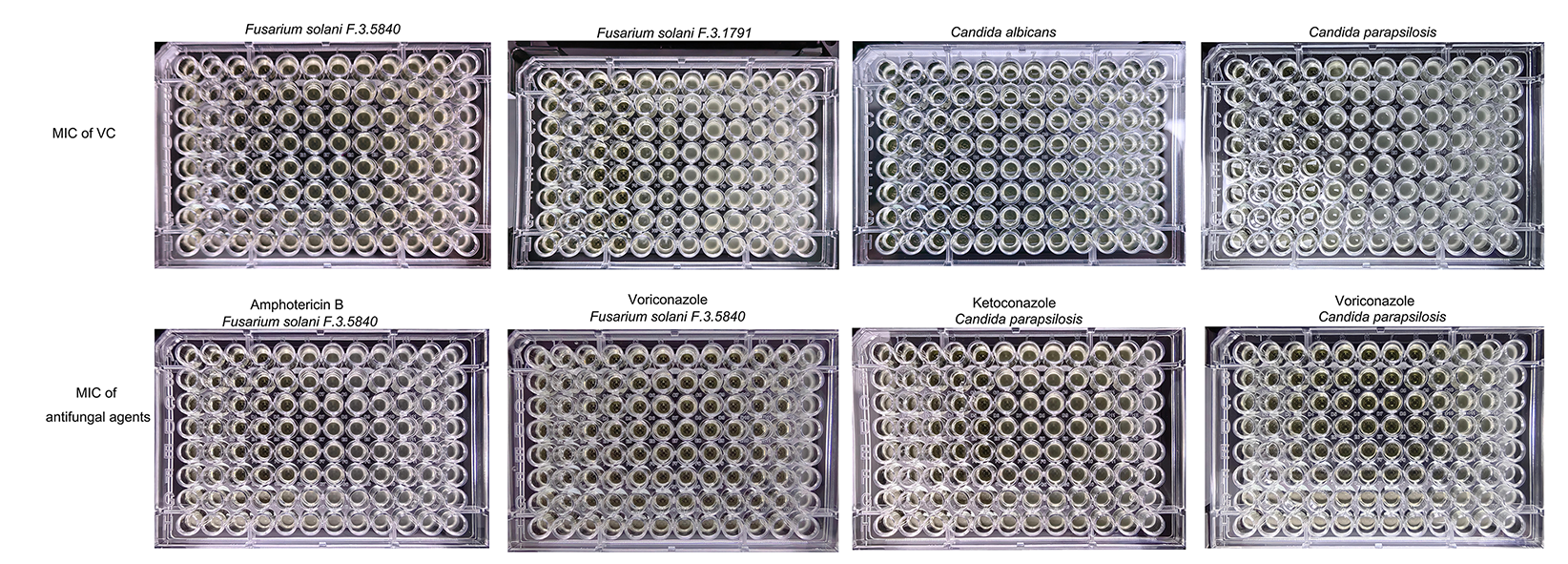

Supplement: Fig. S5 — Representative images of MIC plates. [file aac.01165-25-s0005.tif]
