# Supplementary material for: Vitamin C enhances corneal fungal infection treatment in mice via chemotaxis and anti-inflammation
Source: Antimicrob Agents Chemother. 2025 Nov 28;70(1):e01165-25. doi: 10.1128/aac.01165-25 (PMC12777558; doi:10.1128/aac.01165-25)
Supplement: Supplemental material — Supplemental figure legends. [file aac.01165-25-s0006.docx]

**Supplemental Figure Legends**

Fig. 1 Mouse body weight changes following 7-day different drinking water treatment. Body weight (grams) of mice was measured at day 0 and day 7 in two groups: normal water (H_2_O) and 3 g/L vitamin C (VC) water. Comparison of weight distributions between time points and groups revealed no significant differences, indicating that 3 g/L VC supplementation via drinking water does not affect mouse body weight.

Fig. 2 Typical images of corneal perforation.

Fig. 3 A stepwise gating strategy to identify and quantify neutrophils in mouse corneal single-cell suspensions.Step 1: Gate on "Single Cells" using FSC-A vs. FSC-H to exclude doublets. Step 2: Gate on "Live Cells" using a viability dye (FSV510^-^) to exclude dead cells. Step 3: Gate on "leukocytes" (CD45^+^) from the live cell population. Step 4: Subgate "Neutrophils" as CD11b^+^Ly6G/6C^+^ from leukocytes cells, and quantify the percentage of this subset relative to total single cells.

Fig. 4 Blank control for CFU assay. To verify the stability of the experimental system and the sterility of the surrounding culture environment, parallel control experiments for the CFU assay were conducted. The **Blank** **group** contained only RPMI 1640 medium diluted with 0.01 M PBS, while the **Neutrophil** **group** contained only neutrophils diluted with 0.01 M PBS and RPMI 1640 medium(with the dilution method identical to that described in Section 2.8.2 "CFU Assay" of the main text).

Fig. 5 Representative images of MIC plates. The first row shows the MIC plates of VC against four fungi.The second row shows the MIC plates of antifungal drugs against two fungi respectively.
